# Computer-aided prognosis of tuberculous meningitis combining imaging and non-imaging data

**DOI:** 10.1038/s41598-024-68308-8

**Published:** 2024-07-30

**Authors:** Liane S. Canas, Trinh H. K. Dong, Daniel Beasley, Joseph Donovan, Jon O. Cleary, Richard Brown, Nguyen Thuy Thuong Thuong, Phu Hoan Nguyen, Ha Thi Nguyen, Reza Razavi, Sebastien Ourselin, Guy E. Thwaites, Marc Modat, the Vietnam ICU Translational Applications Laboratory (VITAL) Investigators, Dang Phuong Thao, Dang Trung Kien, Doan Bui Xuan Thy, Dong Huu Khanh Trinh, Du Hong Duc, Ronald Geskus, Ho Bich Hai, Ho Quang Chanh, Ho Van Hien, Huynh Trung Trieu, Evelyne Kestelyn, Lam Minh Yen, Le Dinh Van Khoa, Le Thanh Phuong, Le Thuy Thuy Khanh, Luu Hoai Bao Tran, Luu Phuoc An, Angela Mcbride, Nguyen Lam Vuong, Nguyen Quang Huy, Nguyen Than Ha Quyen, Nguyen Thanh Ngoc, Nguyen Thi Giang, Nguyen Thi Diem Trinh, Nguyen Thi Le Thanh, Nguyen Thi Phuong Dung, Nguyen Thi Phuong Thao, Ninh Thi Thanh Van, Pham Tieu Kieu, Phan Nguyen Quoc Khanh, Phung Khanh Lam, Phung Tran Huy Nhat, Guy Thwaites, Louise Thwaites, Tran Minh Duc, Trinh Manh Hung, Hugo Turner, Jennifer Ilo Van Nuil, Vo Tan Hoang, Vu Ngo Thanh Huyen, Sophie Yacoub, Cao Thi Tam, Duong Bich Thuy, Ha Thi Hai Duong, Ho Dang Trung Nghia, Le Buu Chau, Le Mau Toan, Le Ngoc Minh Thu, Le Thi Mai Thao, Luong Thi Hue Tai, Nguyen Hoan Phu, Nguyen Quoc Viet, Nguyen Thanh Dung, Nguyen Thanh Nguyen, Nguyen Thanh Phong, Nguyen Thi Kim Anh, Nguyen Van Hao, Nguyen Van Thanh Duoc, Pham Kieu Nguyet Oanh, Phan Thi Hong Van, Phan Tu Qui, Phan Vinh Tho, Truong Thi Phuong Thao, Natasha Ali, David Clifton, Mike English, Jannis Hagenah, Ping Lu, Jacob McKnight, Chris Paton, Tingting Zhu, Pantelis Georgiou, Bernard Hernandez Perez, Kerri Hill-Cawthorne, Alison Holmes, Stefan Karolcik, Damien Ming, Nicolas Moser, Jesus Rodriguez Manzano, Liane Canas, Alberto Gomez, Hamideh Kerdegari, Andrew King, Marc Modat, Reza Razavi, Miguel Xochicale, Walter Karlen, Linda Denehy, Thomas Rollinson, Luigi Pisani, Marcus Schultz

**Affiliations:** 1 School of Biomedical Engineering & Imaging Sciences, King’s College London, London, UK; 2 Nuffield Department of Medicine, University of Oxford, Oxford, UK; 3 Oxford University Clinical Research Unit, Ho Chi Minh City, Viet Nam; 4 London School of Hygiene & Tropical Medicine, London, UK; 5 Department of Radiology, Guy’s and St, Thomas’ NHS Foundation Trust, London, UK; 6 Oxford University Clinical Research Unit, Ho Chi Minh City, Viet Nam; 7 The Hospital for Tropical Diseases, Ho Chi Minh City, Viet Nam; 8 University of Oxford, Oxford, UK; 9 Imperial College London, London, UK; 10 King’s College London, London, UK; 11 Ulm University, Ulm, Germany; 12 The University of Melbourne, Melbourne, Australia; 13 Mahidol Oxford Tropical Medicine Research Unit, Bangkok, Thailand

**Keywords:** Tuberculous meningitis, Prognosis, Machine learning, Long short-term memory network, DenseNet, MRI imaging, Prognostic markers, Tuberculosis, Brain imaging, Magnetic resonance imaging, Biomedical engineering

## Abstract

Tuberculous meningitis (TBM) is the most lethal form of tuberculosis. Clinical features, such as coma, can predict death, but they are insufficient for the accurate prognosis of other outcomes, especially when impacted by co-morbidities such as HIV infection. Brain magnetic resonance imaging (MRI) characterises the extent and severity of disease and may enable more accurate prediction of complications and poor outcomes. We analysed clinical and brain MRI data from a prospective longitudinal study of 216 adults with TBM; 73 (34%) were HIV-positive, a factor highly correlated with mortality. We implemented an end-to-end framework to model clinical and imaging features to predict disease progression. Our model used state-of-the-art machine learning models for automatic imaging feature encoding, and time-series models for forecasting, to predict TBM progression. The proposed approach is designed to be robust to missing data via a novel tailored model optimisation framework. Our model achieved a 60% balanced accuracy in predicting the prognosis of TBM patients over the six different classes. HIV status did not alter the performance of the models. Furthermore, our approach identified brain morphological lesions caused by TBM in both HIV and non-HIV-infected, associating lesions to the disease staging with an overall accuracy of 96%. These results suggest that the lesions caused by TBM are analogous in both populations, regardless of the severity of the disease. Lastly, our models correctly identified changes in disease symptomatology and severity in 80% of the cases. Our approach is the first attempt at predicting the prognosis of TBM by combining imaging and clinical data, via a machine learning model. The approach has the potential to accurately predict disease progression and enable timely clinical intervention.

## Introduction

In 2020, tuberculosis (TB) affected 10 million people worldwide and killed 1.5 million^1^. The most threatening form of TB, tuberculous meningitis (TBM), occurs in 2–5% of TB cases^1,2^, causing morphological changes in the brain as a consequence of *Mycobacterium tuberculosis* infection. TBM is currently the leading cause of bacterial brain infection in settings where TB is highly prevalent, affecting particularly children and immunosuppressed individuals, especially those living with human immunodeficiency virus (HIV)^2^.

Defining the prognosis of TBM can be challenging, particularly when impacted by other comorbidities and underlying illnesses such as HIV^3,4^. Therefore, it is essential to develop approaches that accurately and quantitatively stage and predict disease progression, thereby enabling timely clinical interventions. TBM severity and outcomes are currently assessed using clinical scales, such as the TBM Medical Research Council (MRC) grade^5^ , which defines severity, and the Modified Rankin Scale (mRS)^6,7^, which evaluates the neurological outcomes of the subjects. However, these clinical tools do not accurately characterise or predict disease progression.

Recently, Evans et al.^8^ and Thao et al.^9,10^ developed accurate methods to identify patients with TBM at a high risk of severe complications with poor outcomes requiring long-term rehabilitation. Their prognostic models focused on the use of clinical assessments such as the Glasgow Coma Scale (GCS) or the MRC grade that characterise the disease severity^9,10^. Such models often rely on qualitative assessments, which hampers the prediction results and make them prone to user-dependence. An alternative to clinical scales is the use of brain imaging, using Magnetic Resonance Imaging (MRI) and Computer Tomography (CT), which may be more reliable tools at characterising and predicting the severity and progression of TBM.

Machine learning (ML) approaches have been used successfully to study disease progression, particularly for neurological diseases and disorders^11^. Many models combine individual demographic profiles, existing comorbidities, and clinical assessments with imaging data. Deep-learning models (DL) enable extraction, encoding, and modelling of features from multi-source data, removing the need to manually engineer features (i.e., handcraft features)^11,12^, and often outperform traditional ML models. Deep convolutional networks, such as ResNet and DenseNet^13^, are a popular options to diagnose and characterise the evolution of dementias^14,15^, predict the outcome and survival of individuals with brain tumours^16^, or even predict the brain age of neurologically and radiologically normal individuals^17,18^. However, to date, ML and DL approaches have not been applied to TBM, combining imaging and other clinical data.

Therefore, we developed a tailored model to (1) extract brain imaging features to characterise TBM disease severity, and (2) predict their prognosis using both imaging and clinical data. Our model is composed of a DenseNet block to process the input images, followed by a time-series model inspired by the models used for forecasting^19^, to predict a patient’s condition evolution. Unlike available models designed for disease prognosis, our model does not require a constant number of observations across individuals or equally spaced time points. To accommodate missing data, particularly missing imaging data, we defined an optimization framework that accounts for the distance the missing sample and the acquired equivalent data. As a result, our model can generate predictions for any time-point during the disease progression, regardless of the latest information acquired. This feature is particularly useful in disease progression models where data are sparse or missing.

To the best of our knowledge, our model is the first attempt to predict TBM outcome using imaging data, in an automated end-to-end approach robust to missing data.

## Methods

### Study participants and data

The data used in this study was acquired from two prospective longitudinal studies run by the Oxford University Clinical Research Unit (OUCRU) in Vietnam. The participants were enrolled into two clinical trials to assess the benefit of adjunctive dexamethasone in HIV-positive (HIV-p) and HIV-negative (HIV-n) adults (> 17 years) with TBM (RCTs identifiers NCT03092817 and NCT03100786, respectively). Both trials were approved from local and national ethics and regulatory authorities in Vietnam and Indonesia and from the Oxford Tropical Research Ethics Committee in the United Kingdom, as described in online Appendix (p. 3) and in previous studies^20,21^. Details of the entry and exclusion criteria, treatment and follow-up of participants are described elsewhere^20,21^. Briefly, participants were recruited from the Hospital for Tropical Diseases in Ho Chi Minh City, Vietnam, with a clinical diagnosis of TBM (at least 5 days of meningitis symptoms and cerebrospinal fluid abnormalities), with anti-tuberculosis chemotherapy either planned or started by the attending physician. Written informed consent to enter the trial was obtained from all the participants or a relative if they were incapacitated, with subsequent consent from the patient when capable. All analyses in this study were performed in accordance with the relevant guidelines and regulations defined in both trials.

Participants were subsequently classified as having definite, probable, or possible tuberculous meningitis, following published criteria^4^. Patients were ineligible if another brain infection was suspected, if > 6 consecutive days of anti-tuberculosis chemotherapy or > 3 consecutive days of systemic corticosteroids were received before enrolment, or if systemic corticosteroids were considered mandatory or contraindicated for any reason. Participants underwent clinical assessments at baseline, at days 3, 7, 10, 14, 21, and 30, and monthly until month 12. Assessment included Glasgow coma score, modified MRC severity grade, MRI brain imaging, and details of clinical and adverse events and other interventions. Neurological outcomes were assessed by the modified Rankin score (mRS). The results from the ACT-HIV trial (in HIV-positive adults) were recently published^22^, the LAST-ACT trial will not complete participant follow-up until May 2024 and participants and investigators remain blind to the treatment allocation (dexamethasone or placebo).

We collected longitudinal information from 216 consecutive participants enrolled on the trials, three-dimensional (3D) T1-weighted GRE brain MRI scans acquired with magnetization-prepared rapid acquisition (T1w—MPRAGE) pulse sequence (parameters: Repetition Time [TR] = 1920 ms; Echo Time [TE] = 5.1 ms; Inversion Time [IT] = 1100 ms; Flip Angle = 15°; Field Of View [FOV] = 168 × 256 × 160 voxels; Spatial Resolution = 1.00 mm × 1.00 mm × 1.15 mm), and non-imaging data. The inclusion criteria for the current study were: (1) having at least one imaging time-point, (2) having baseline clinical evaluation and imaging data within 30 days of study entry, and (3) having at least one follow-up clinical assessment during the trial (24 months for HIV-n and 12 months for HIV-p participants). Individuals who lacked imaging data or only underwent a baseline assessment were excluded from participation. Further details regarding patients’ inclusion criteria in the study are available in online Appendix p.5, Figure S1. Clinical assessments included cerebrospinal fluid (CSF) examination, presence of cranial nerve palsy or other focal neurological deficit, temperature, HIV status, TBM MRC grade^5^, mRS^6^ and demographic information, such as gender, age, and body mass index (BMI). Not all data was available for all the time points, with missing at random variables. Brain MRI images were acquired at three different time points as per protocol^20,21^: enrolment time, two months, and one year of follow-up. Additional MRI images were obtained according to clinical need.

### Disease prognosis and longitudinal prediction

**Figure 1:**
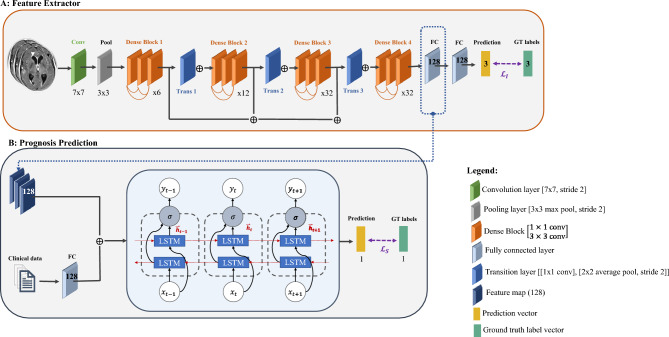
Model scheme for individual prognosis prediction. (**A:**) Feature extractor block, a DenseNet architecture backbone^13^, extracts and encodes the imaging features. The optimisation of this first block is achieved by the contribution of the imaging loss (${\mathcal{L}}_{I}$) to the final loss of the model, which assesses the accuracy of the predictions of TBM grade for each MRI scan individually. (**B:**) Prognosis prediction uses both the clinical (numerical vector) and imaging features (feature maps obtained by the feature extractor block) and uses a bilateral LSTM to predict the mRS scale for the next time-point in the sequence via optimisation of loss of the sequence (${\mathcal{L}}_{S}$). The full sequence per individual is assessed. FC: fully connected layer. GT: Ground-true labels. Conv: Convolution layer. Trans: Transition layer. LSTM: Long short-term memory model.

The proposed model consists of an end-to-end approach designed to model the longitudinal brain and clinical changes that are associated with the symptoms of the disease and their severity. The mRS grade, which assesses neurological disability, is used as a surrogate measure of outcome severity, hereafter denominated as the response variable. Figure 1 describes the full model where (A) corresponds to the imaging features encoding, followed by (B) the sequence modelling and prediction.

In detail, given a dataset $D={\{{{\varvec{X}}}_{i},\boldsymbol{ }{{\varvec{Y}}}_{i}\}}_{i=1}^{N}$, for participant $i\in \left[1, N\right],$ ***X***_***i***_ encodes the individual features used to explain the response variable ***Y***_***i***_, with ${{\varvec{X}}}_{{\varvec{i}}}={\{{[{{\varvec{x}}}_{i,t,f}]}_{t=1}^{\text{\rm T}}\}}_{f=1}^{F}$ for time-point *t* and feature *f*. The T1w were manually assessed for artefacts and excluded if necessary. As a pre-processing step, the T1w scans were rigidly spatially aligned to the Montreal neurological institute (MNI) brain atlas^23^ space using the *NiftyReg* open-source software^24^ to ensure comparability across subjects, with the resulting images with dimensions 180 × 210 × 180 voxels and resolution 1 mm × 1 mm × 1mm.

Each time-point considered by the model included a set of 15 clinical features (Table S2, online Appendix p.3) and a T1w image, registered to MNI space. However, due to different in acquisition frequencies between the clinical and imaging data and the presence of missing data, this varied across subjects and time-points.

The following sections describe the imaging features encoding and individual disease progression prediction.

*A: Features Encoding*

To extract quantitative imaging features from the T1w, our proposed model used a DenseNet backbone (Fig. 1A). We selected this architecture because it ensures maximum information flow between different layers while requiring fewer parameters than traditional convolutional networks, as it does not re-learn redundant feature maps^13^. A network with fewer parameters, as this one, is also easier to train and more robust in regimes with small sample sizes. Therefore, this architecture is particularly relevant for medical imaging problems, where data is often scarce and heterogeneous.

The model considered as input the T1w images, and it was optimised to predict the TBM MRC grade acquired concurrently with the MRI, by the clinical team. Typical image transformations were applied using Medical Open Network for AI (MONAI)^28^, specifically image intensity scaling between 0 and 1, and image resize to 128 × 128 × 128 voxels, with 1 mm^3^ resolution, to reduce image size and memory usage by the model. Further transformations for data augmentation purposes were applied during the model training to increase model generalisation, as described in online Appendix (Table S3, page 4). The model architecture emulates the DenseNet-169 architecture^13^, as described in Fig. 1. Our classification layer did not include a global average pool like the original DenseNet. Instead, we included two fully connected layers (output size: 128), followed by a *softmax* function with output size three, corresponding to the three possible values of the TBM MRC grade.

The parameters of the feature extractor (block A, Fig. 1) were optimised via the contribution of the weighted cross-entropy loss^25^ (${\mathcal{L}}_{I}$) to the final loss, whose weights were estimated based on the frequency of each class on the training set.

*B: Prognosis model—sequence prediction*

The prediction of the individual status during the disease is addressed by a time-series model (Fig. 1B), where a long short-term memory (LSTM) model is used^26^. The LSTM models are gated recurrent neural networks with feedback connections designed to process sequences of data. Furthermore, these models use the information of the present time step (short-term) while considering the relevant information from the complete sequence. Therefore, LSTM models can be of interest to analyse temporal data as disease progression time series.

Our prognosis prediction model considered as input the clinical and the imaging-derived features to predict the response variable—mRS scale. The model is composed of an initial fully connected layer (multi-layer perceptron—MLP) in which the clinical features were inputted to extract a relevant feature vector. Due to the non-linear nature of this layer, we were able to capture the complex relationships between the 15 clinical features and to output a high-dimensional feature space (output size = 128), comparable to the imaging-derived feature space.

The obtained clinical feature map was then concatenated with the imaging feature map and used as input in a bilateral LSTM^19^ (hidden nodes = 256 per layer, layers = 5, weights initialised to zero). Note that the bilateral LSTM learns the inputted sequence, and simultaneously learns how to reverse it. This property promotes the learning of the disease biomarkers’ evolution and the processes leading to the individual states.

The prognosis prediction model was designed as a regression model with a 1D output, and its parameters were optimised with the contribution of the sequence loss function (${\mathcal{L}}_{S}$) to the final loss.

#### Loss function

The optimisation of the proposed model was achieved by the minimisation of the loss function:

$$\mathcal{L}={{\beta }_{I}\mathcal{L}}_{I}+{{\beta }_{S}\mathcal{L}}_{S}$$

where ${\beta }_{I}$ and ${\beta }_{S}$ correspond to the weight of the imaging loss and sequence loss respectively. The imaging loss is then encoded as:

$${\mathcal{L}}_{I}:=\text{log}H \left(p,q\right)= - \sum_{t=1}^{T}\sum_{k=1}^{K}{\omega }_{k}p\left({x}_{k,t}\right)\text{ log}(q({x}_{k,t}))$$

where H(.) represents information entropy, $p\left(.\right)$ represents the true distribution of the function, $q\left(.\right)$ represents the predicted distribution, ${\omega }_{k}$ encodes the weight of the loss for the class $k\in \{1, 2, 3\}$ in the TBM grade. Finally, the sequence loss is defined as:

$${\mathcal{L}}_{S}= \frac{1}{T} \sum_{t}^{T}{{{{\varvec{\omega}}}_{\delta }{\odot{\varvec{\omega}}}_{c}\odot \Vert {x}_{ t}-{\widehat{x}}_{t}\Vert }_{2}}+\frac{1}{T-1}\sum_{t}^{T-1}[{\Vert ({{\widehat{x}}_{t+1}-\widehat{x}}_{t})\Vert }_{2}-\sum_{c}^{C}{\Vert ({{x}_{t+1}-x}_{t})\Vert }_{2} \times \text{exp}(-{{\Vert c-{x}_{t}\Vert }_{2}})]$$

where ${x}_{ t}$ represents the ground truth label, the ${\widehat{x}}_{t}$ encodes the predicted label, ${{\varvec{\omega}}}_{c}$ is a vector of the weights of each class $c\in [0, 5]$ encoding the mRS scale, and ${{\varvec{\omega}}}_{\delta }$ is a vector of relevance of the MRI to the current time-point estimated using a gaussian kernel function ${{\varvec{\omega}}}_{\delta }=\text{exp}(-\frac{{\updelta }^{2}}{2*{\upsigma }^{2}})$ with $\updelta$ encoding the time distance to the nearest real image as $\updelta \in [0,\Delta ]$. This term corrects the bias introduced by imputed data, making our approach more robust to missing information. The second term encodes the difference between the predicted class at time point *t* and the next time point to enforce the model to learn changes in prognosis during disease course.

### Experiments

We assessed the performance of the proposed model using the prospective data from the OUCRU trials, described in study participants and data section. All models were optimised using the Joint Academic Data Science Endeavour (JADE) II cluster, with NVIDIA Tesla V100 32 GB GPUs^27^. The models were trained on 2/3 of the available data and the remaining 2/6 were used for validation and testing (1/6 each). Subjects were randomly assigned to the training, validation or testing sets, including all their time points to avoid double-dipping. The models were trained for 200 epochs using the defined and fixed training and validation sets. The best-performing model was chosen based on its performance on the validation set. To prevent biases during model training, the order of subjects in both the training and validation sets was shuffled for each epoch.

We conducted two sets of experiments to independently evaluate (A) the performance of the feature extraction and the clinical relevance of the imaging feature maps, and (B) the accuracy of the prognosis model.

*A: Features extractor: imaging feature maps*

We predicted the TBM MRC grade longitudinally for all individuals and time-points containing both clinical and imaging data, using the feature extractor model (Fig. 1, block A). Performance metrics for multiclass tasks were computed to assess model efficiency. We also conducted a sensitivity analysis to assess the performance of the model in different populations: HIV-p versus HIV-n.

To investigate the possible clinical meaning of the optimised imaging features, we computed occlusion sensitivity maps for model predictions for both HIV-p and HIV-n individuals. These maps encode how the probability of a given prediction changes in specific sections of the MRI imaging. They can bring insights regarding which brain regions have a higher impact on the differentiation of the severity stages of the disease. We used the MONAI^28^ to generate the occlusion-sensitivity maps.

*B: Prognosis model—sequence prediction*

The missing clinical data were imputed using the multi-imputation chain equations technique (MICE) with the k-mean nearest neighbour, except for TBM MRC grades and mRS score. A missing TBM grade was not imputed leading to the participant and/or time-point exclusion. mRS score was imputed using additional clinical information, when available, as described in Table S1 (online Appendix p.3). When missing, the imaging data is imputed using the nearest imaging time-point, and its relevance to the response variable prediction is subsequently pondered according to its distance to the time-point. I.e., during the training of the model, the distance between the MRI scan and the predicted time-point is used both as a clinical feature and a relevance factor considered in the loss function for the prognosis prediction block (Fig. 1, B), while during inference this distance is only considered as a clinical feature.

We evaluated the performance of the prognosis model by assessing the correctness of the mRS score longitudinally (distribution of mRS score in testing set available in online Appendix, pp.4–5, Table S4, Figure S2). Note that the predicted label was a continuous variable; therefore, the predicted label value was rounded to the closest integer for a fair comparison with the ground truth labels (categorical scale). We conducted this evaluation on the full population and, as before, used standard multiclassification metrics to evaluate the accuracy of the results.

We compared the model performance with a previous study on TBM prognosis^9^. In this study, the authors used a multivariate model embedded with feature selection approach to identify the best clinical features to predict survival during a 9-month follow-up period, where the MRC grade and GCS score proven to be useful predictors of individual outcome. For a fair comparison, we trained the multivariate LASSO model using the covariates defined in the study^9^. The model was optimised using the training data with nested cross-validation to select the best alpha parameter, and subsequently evaluated on the testing set.

To assess the respective predictive power of the clinical and imaging features to the prognosis prediction, we evaluated the performance of the proposed model using: (1) only the clinical data, using the LSTM only (clinical model—*Cm*), and (2) only the imaging data extracted using the DenseNet backbone feature extractor and the LSTM (imaging model—*Im*).

We performed a sensitivity analysis on the HIV-p and HIV-n populations to assess the predictive value of the several models on different phenotypes of the disease.

Lastly, we further assessed the performance of the model in predicting the disease dynamics over time. I.e., we empirically grouped the mRS scale into three classes according to the subject status evolution across two consecutive time-points: decreased (decreasing mRS, improvement of subject health status), increased (increasing mRS, worsening of symptoms), and stable (frequency of disease progression labels across time points available in online Appendix, p.4 Table S4). The model was not specifically trained for this task, consisting of a post-processing analysis of the results of the sequence prediction.

#### Evaluation metrics

The performance of the optimised models was assessed using multi-class classification metrics, namely balanced accuracy (bAcc) defined as the average of recall obtained on each class, Matthew correlation score (MCC) that evaluates the quality of multiclass classifications as a correlation score between  − 1 and + 1 (inverse prediction and perfect prediction, respectively), weighted recall and weighted precision, with TP, TN, FP and FN encoding true positive, true negative, false positive and false negative, respectively for class *k* and *n* representing the number of samples for class *k.*

$$bAcc= {\sum }_{k=1}^{K}\frac{T{P}_{k}+T{N}_{k}}{(T{P}_{k}+T{N}_{k}+F{P}_{k}+F{N}_{k})}$$

$${\text{MCC}}={\sum }_{k=1}^{K}\frac{T{P}_{k}\times T{N}_{k}-F{P}_{k}\times F{N}_{k}}{\sqrt{\left(T{P}_{k}+F{P}_{k}\right)\left(T{P}_{k}+F{N}_{k}\right)\left(T{N}_{k}+F{P}_{k}\right)\left(T{N}_{k}+F{N}_{k}\right)}}$$

$$Weighted \,recall= {\sum }_{k=1}^{K}\frac{1}{{n}_{k}}\frac{T{P}_{k}}{(T{P}_{k}+F{N}_{k})}$$

$$Weighted\, precision= {\sum }_{k=1}^{K}\frac{1}{{n}_{k}}\frac{T{P}_{k}}{(T{P}_{k}+F{P}_{k})}$$

### Role of funding source

None of the funders had a role in study design, data analysis or data interpretation.

### Ethics

The trials (RCTs, identifiers NCT03092817 and NCT03100786) received ethical approvals from Hospital for Tropical Diseases, the Vietnam Ministry of Health, and the Oxford Tropical Research Ethics Committee, as described^20,21^.

## Results

The full dataset comprised 216 subjects from the OUCRU cohort, including 73 HIV-p and 143 HIV-n (Table 1). We assessed whether significant differences existed between the two subpopulations (i.e., HIV-p versus HIV-n), for age, BMI, illness duration and number of assessments, including clinical and imaging evaluations. A Mann–Whitney test demonstrated that the two subpopulations significantly differ in the demographic features available (Table 1), with *p* value < 0.01 for age, BMI, follow-up duration and number of clinical assessments after correction for multiple comparisons using Bonferroni’s approach (number of tests = 3).

**Table 1:** Demographic information of the study population.

		HIV positive		HIV negative	
Number		73		143	
Age (years)*		31.0 [24.0; 36.0]		35 [27.0; 46.0]	
BMI (kg/m2)*		18.86 [16.52; 20.93]		20.20 [18.75; 22.22]	
Sex (percentage of Males)		59 (80.8%)		97 (67.8%)	
Follow-up duration (days)*		640.0 [122.0; 735.0]		362.0 [336.0; 366.0]	
Diagnostic category (percentage)	Possible TBM	9 (12.3%)		22 (15.4%)	
	Probable TBM	4 (5.4%)		14 (9.8%)	
	Definitive TBM	60 (82.2%)		72 (50.3%)	
	Unknown (scores not available at the time of the study)	0 (0.0%)		35 (24.5%)	
Number of clinical assessments during trials (12 and 24 months, respectively)		18.0 [12.0; 20.0]		14.0 [11.0; 16.0]	
Number of clinical assessments with imaging data		5.0 [3.0; 6.0]		4.0 [2.0; 5.0]	
Death (percentage)		9 (12.3%)		4 (2.8%)	
Years since HIV diagnosis		1 [0.0; 3.0]		N/A	
Clinical Evaluations		Baseline	Follow-ups	Baseline	Follow-ups
TBM grade, number of time-points (ratio)	TBM grade = 1	36 (0.49)	36 (0.49)	69 (0.48)	69 (0.48)
	TBM grade = 2	34 (0.47)	34 (0.47)	70 (0.49)	70 (0.49)
	TBM grade = 3	3 (0.04)	3 (0.04)	4 (0.03)	4 (0.03)
mRS scale, number of time-points (ratio)	mRS score = 0	18 (0.25)	44 (0.60)	44 (0.31)	109 (0.76)
	mRS score = 1	14 (0.19)	46 (0.63)	52 (0.36)	109 (0.76)
	mRS score = 2	14 (0.19)	29 (0.39)	18 (0.13)	35 (0.25)
	mRS score = 3	7 (0.10)	20 (0.27)	10 (0.07)	23 (0.16)
	mRS score = 4	4 (0.06)	13 (0.18)	5 (0.04)	10 (0.07)
	mRS score = 5	16 (0.22)	19 (0.26)	14 (0.10)	20 (0.14)

Median values and interquartile range [IQR] are presented for age, body mass index (BMI), duration, and years since HIV diagnosis. Number of samples with a given TBM grade and mRS reported at baseline and follow-up time-points (multiple values per subject) and percentage of samples with this value in parentheses. Gender, death, TBM grade and mRS scale are presented as the number of subjects per subgroup and their ratio from each population. Frequency of time points per mRS score is available in the online Appendix, Table S4. N/A: not applicable.

*Ecodes significancy with p-value < 0.01 evaluated with Mann–Whitney statistical test (after correction for multiple comparisons, number of tests = 3).

### Features extractor: imaging feature maps

We assessed the performance of the model in the extraction of quantitative imaging features to describe the TBM severity by predicting MRC TBM grade. Table 2 summarises the performance of the feature extractor. Overall, the feature extractor model was able to accurately predict the TBM grade from imaging data (balanced accuracy of 0.96 and MCC of 0.89).

**Table 2:** Model performance when predicting the TBM grade.

	Balanced accuracy	MCC	Recall weighted	Precision weighted
All Data	0.96	0.89	0.94	0.94
HIV-p patients	0.88	0.79	0.89	0.91
HIV-n patients	0.98	0.94	0.96	0.97

Model evaluation in the testing set using the best-performing model in the validation set. All data refer to the full population, including HIV-p and HIV-n. MCC: Matthew correlation score.

**Figure 2:**
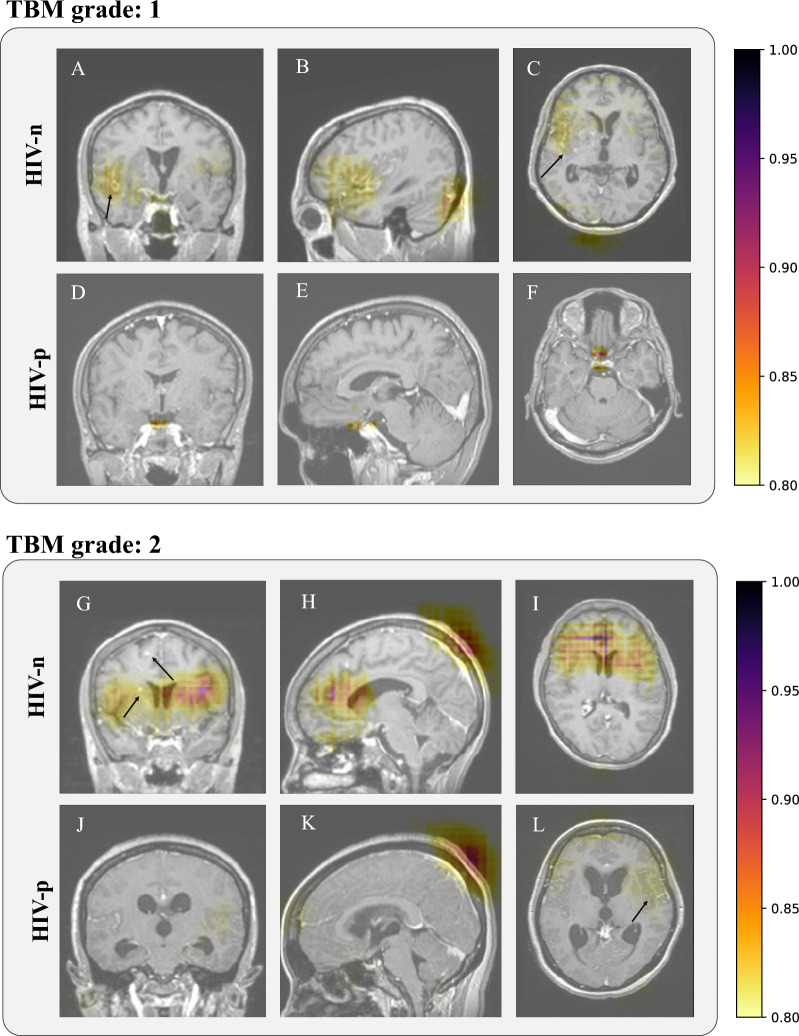
Occlusion sensitivity imaging feature maps for correctly classified patients for coronal, sagittal and axial views (left to right, respectively). (**A**–**F**): Feature maps for class 0 (TBM grade 1), for HIV-n (**A**–**C**) and HIV-p (**D** to **F**). **G**–**M**: Feature maps for class 1 (TBM grade 2), for HIV-n (**G** to **I**) and HIV-p (**K** to** L**). Colormap encodes the relevance of features extracted from the MRI scan, with red encoding highly relevant brain areas for the predicted class and white encoding lower relevance. The occlusion sensitivity maps ranged from 0 to 1, illustrating the probability of each region to highly impact the classification. Black arrows point existent morphological lesions associated to TBM: (**A**)—tuberculomas and adjacent meningitis, (**G**)—tuberculomas, (**C**, **L**)- vasculitis. Colourmaps were normalised between 0 and 1, with the visualisation threshold set to 0.8.

We computed the occlusion sensitivity maps for the patients correctly classified for TBM grades equal to 1 and 2 (Fig. 2A–F and G–L, respectively). Due to the small sample size for TBM grade equal to 3, we did not further evaluate feature relevance on these patients. Occlusion sensitivity maps were computed separately for HIV-p and HIV-n subjects. Overall, the model identified as relevant areas for classification existing lesions as tuberculoma (Fig. 2A, G–H, black arrows), vasculitis (Fig. 2C, J, L) and hydrocephalus (Fig. 2J–L). Additionally, the sinus vein was identified as relevant in more severe diagnosis (TBM grade equal to 2), suggesting the relation between this area and more worst outcomes. No evident differences were found across populations (HIV-p versus HIV-n), in terms of lesion localisation, with a predominance of vasculitis in HIV-p patients and tuberculomas in HIV-n. However, such findings would need to be validated in larger populations. Note that, despite the model identified as most relevant the coloured regions, the full image contributed for the classification model predictions leverage all the contextual information.

### Prognosis model—sequence prediction

**Figure 3:**
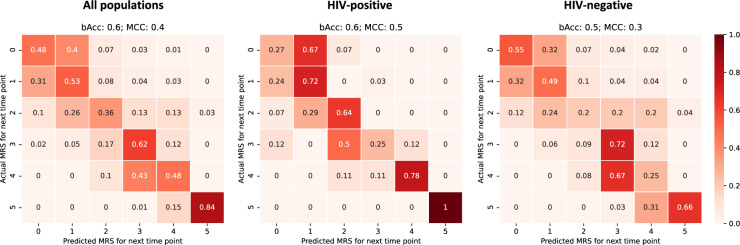
Confusion matrix of prognosis prediction on the testing set. The confusion matrix is computed considering all time-points of each sequence for the full population, including both HIV-p and HIV-n (left panel), HIV-p (centre) and HIV-n (right panel). bACC: Balanced accuracy. MCC: Mathew correlation score.

We assessed the performance of the proposed prognosis model when predicting the mRS scale longitudinally, for the full population (Fig. 3A), and for each sub-group independently (HIV-p and HIV-n, Fig. 3B and C respectively, distribution of labels for each population available in online Appendix p.4, Table S4). The performance metrics are overall higher in the prognosis of HIV-p patients, for which MCC is 0.5 and balanced accuracy is 0.6 (random guessing per class is approximately 0.17, when in the presence of a balanced number of samples per class, further details about class imbalance available in online Appendix p.4, Table S4), with a higher rate of misclassification for mRS equal to 0 (no symptoms). Such a result is linked to a higher prevalence of brain morphological changes linked to the HIV co-occurrence. Differently, for HIV-n patients the model performance scores are lower for intermediate stages of the disease (mRS equal to 2 and 4), due to the small sample size for such classes and the less evident imaging features. Nevertheless, the model was able to accurately predict the patient prognosis when trained on both populations, with a balanced accuracy of 0.6 (MCC of 0.4), demonstrating its robustness to individuals with different comorbidities and TBM phenotypes.

**Figure 4:**
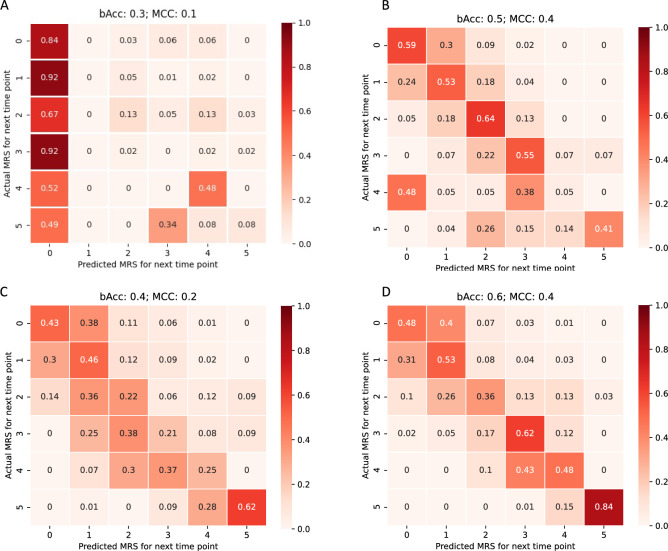
Confusion matrix of prognosis prediction on the testing set. The confusion matrix is computed considering all time-points of each sequence for the full population, including both HIV-p and HIV-n. (**A**): Multivariate prognosis model (MV-PM) using clinical features^9^. (**B**): Prognosis model using clinical features only. (**C**): Prognosis model using imaging features only. (**D**): Proposed prognosis model using both imaging and clinical features. bACC: Balanced accuracy. MCC: Mathew correlation score.

To better understand the added value of the different data modalities (imaging, clinical and combined features), we assessed the performance of our approach when using the different combinations of features (Fig. 4, Table S5, online Appendix p.4), and we compared it with the existent TBM prognosis model: MV-PM model. The results suggest that the MV-PM model (Table S5) is unable to correctly identify disease progression.

Our model, using clinical data only (*Cm)*, accurately predicts TBM outcome, anticipating mild to moderate outcomes (Fig. 4B, Table S5 online Appendix p.4). These results demonstrate that clinical features, such as CSF count and GCS are sufficient to anticipate patient’s clinical outcomes when presenting with less severe forms of TBM, failing however in predicting higher mRS scores. The models leveraging the imaging data (imaging only–*Im–*and imaging and clinical–*C&I*) show a good prognostic performance, particularly showing a high accuracy in predicting severe outcomes (mRS > = 4) (Fig. 4, C and D). Such results suggest that imaging features are insufficient to accurately predict less severe outcomes (mRS < = 3) but are essential for the anticipation of severe outcomes.

It is worth noting that using only clinical data resulted in comparable precision with models including imaging data (0.62 *versus* 0.49 and 0.61 for *Cm*, *Im* and *C&I*, Table S5, online Appendix p.4). However, the prognosis recall is higher for the model considering both data (0.54 *versus* 0.53 for *C&I* and *Cm* respectively), and the prognosis of HIV-n shows a higher recall and precision for the model leveraging both information (recall: 0.524 *versus* 0.490, and precision: 0.593 *versus* 0.514, for *C&I* and *C*, respectively). This suggests that imaging features can introduce bias when characterizing HIV-p population, particularly for less severe prognosis (Figs. 3, 4, Table S5, online Appendix p.4), where imaging features seem to lead to an overestimation of the mRS scale.

**Figure 5:**
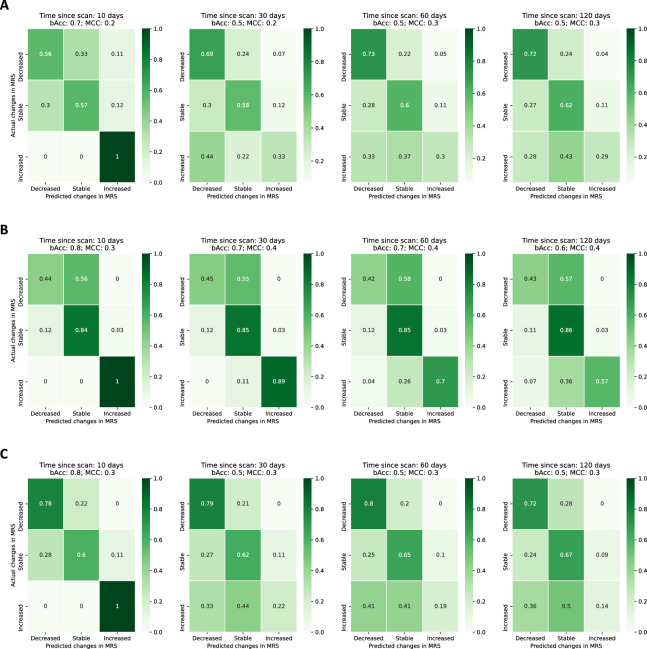
Confusion matrix of disease progression on the testing set given imputed scans. The confusion matrix is computed considering all time-points of each sequence for the full population, including both HIV-p and HIV-n. Increased: the mRS scale increased from the previous timepoint (worsening of patient condition, rise of disease severity). Stable: the mRS scale did not change when compared with the previous timepoint. Decreased: the mRS scale decreased from the previous timepoint (improvement of patient condition). (**A**): Model trained with clinical data only. The time to scan is measure from the available scan as per the models using imaging, even if not used. (**B**): Model trained with imaging only. (**C**): Model trained using imaging and clinical data.

We used predicted prognostic labels to assess the performance of our model in detecting changes in prognosis during the disease course as a consequence of individual responses to disease progression and/or treatment. For that, we categorised the observed differences in mRS score and compared them with the estimated differences from the predicted scores (frequency of observed differences used as ground-true detailed in the online Appendix, p.4, Table S4). Figure 5 shows how well the model captured the subject's progression over the entire sequence when trained using clinical data (Fig. 5A), only imaging data (Fig. 5B) and both imaging and clinical data (Fig. 5C). We also evaluated the impact of the distance (in days) to the last acquired scan when predicting mRS score. Our model was effective in predicting status changes within 10 days of the scan, with an accuracy of 0.8 for models relying on imaging data (0.7 of balanced accuracy for the clinical model), suggesting that the model is more accurate in identifying changes in prognosis when leveraging recently acquired imaging data. Conversely, our approach is less accurate in predicting changes in status when the last scan was acquired more than 30 days from the time of prediction. Furthermore, the model using only imaging data showed a higher performance in detecting the worsening of the patient's status, suggesting that imaging features have the potential to indicate worse outcomes, even when the scan was acquired more than 60 days ago, while the model leveraging clinical and imaging data is more accurate in predicting recovery. Overall, the results suggest that the predictive power decreases with distance to the scan, suggesting that contemporary imaging is needed to accurately anticipate changes in patients’ outcome.

## Discussion

This study presents a novel prognostic model for TBM using clinical and imaging longitudinal data. The proposed approach successfully predicted the mRS score for un-seen time points with a balanced accuracy of 0.6 and an MCC of 0.4 when predicting the disease progression across patients. The results suggest that the imaging and clinical data can help predict the clinical evolution of TBM, specifically the identification of the worst outcomes. However, the model is less accurate in identifying the intermediate stages of severity, where the data was scarcer, and the disease phenotype less extreme (mRS grade 2 and 4). Nonetheless, the model was able to identify changes in individuals’ prognosis during the disease with an accuracy of 0.8, suggesting that even when inaccurate in predicting the actual mRS value, the model detected possible clinical alterations. Such results could be of interest in clinical context to anticipate possible adverse events that could delay or impact recovery.

The previously developed prognosis model^9^ using clinical features was outperformed by our approach when using only clinical features, such as CSF biomarkers and GCS, suggesting that leveraging the temporal relationship of some clinical features improves the prediction of TBM outcomes, as the model proposed by Thao et al.^9^ did not consider the interaction between multiple time-points. By adding both the clinical and imaging features, we demonstrated that the imaging features make use of additional clinical features such as CSF analysis and the patient’s clinical assessment, further improving the prediction of the disease outcome. As a result, our study demonstrates that brain imaging data can help refine the prognosis of TBM.

To assess the influence of HIV on disease severity and progression, we evaluated the sensitivity of the model predicting the mRS score in HIV-positive and HIV-negative populations. The model combining clinical and imaging data performs similarly across all populations (HIV-p, HIV-n, and all), which suggests that it is robust for the TBM prognosis, and it successfully learned features that characterise the distinct progression of both populations. These results show the potential to use our proposed model defining the prognosis of TBM on populations with co-occurring illnesses, without specific parameter tuning. The model optimised using imaging information alone was demonstrated to be less accurate in the prognosis of HIV-p, suggesting that the imaging features are more ambiguous for this population, and more data is needed to conveniently train and assess model performance. For both populations, our model was highly accurate in the prognosis of outcomes changes during the disease if using recently acquired imaging data, suggesting that the performance of the models relying on imaging data is highly impacted by the time of MRI acquisition. Hence, our imputation strategy could have highly impacted the performance of the imaging models. Future work should focus on the developing of new methods for imaging data imputation.

We also investigated the relationship between the learned imaging features and the TBM grade to understand whether affected brain regions or lesions are specific to different stages of the disease. The results obtained via the occlusion sensitivity analysis demonstrated that the model has correctly identified typical TBM lesions for both populations. Also, the brain areas identified in our analysis are consistent with previously reported areas affected by TBM^29–33^, with a high prevalence of hyperintensities caused by inflammation in the basal ganglia (particularly, the thalamus)^33^, and hydrocephalus with enlargement of ventricles^30^. Similarly, the model successfully identified tuberculomas as highly relevant features, regardless of their size, demonstrating the ability of the model to identify the different types of lesions associated with TBM, and therefore its flexibility and robustness to the heterogeneity of TBM brain lesions across subjects. However, unexpected regions such as pituitary gland (Fig. 2E) and scalp were identified as relevant for model predictions (Fig. 2H–K). No clinical meaning were associated with this specific brain regions. Despite these promising results, the prediction of the TBM grade is more accurate for the HIV-n patients, suggesting that the imaging features were less descriptive for more heterogeneous forms of the disease such as in immunosuppressed patients. Also, the sample size for the HIV-p population is smaller, which might have impacted the model training and thus performance. Therefore, the obtained clinical features may be impacted by the model performances. These results should be confirmed in a larger population of severe cases (those with TBM MRC grade 3) to evaluate model performance on those with extensive morphological brain changes.

### Strengths and limitations

The primary strength of our work is the novel approach to defining TBM prognosis. Our study is the first attempt to model the progression of such a heterogeneous disease using imaging and clinical data, providing a step towards a new prognostic tool. Moreover, the proposed model leverages the ability of new AI tools to extract imaging features without clinical assumptions that could bias and neglect relevant brain morphological changes associated with TBM severity and progression. Our model is also robust to missing data and different numbers of time points per patient, which is of utmost importance in the clinical context, where data is not often acquired uniformly across patients.

Our study also benefits from a unique sample with imaging and clinical data acquired in a longitudinal fashion, including subjects with specific comorbidities (HIV positive). Therefore, this is an outstanding opportunity to study TBM progression under different conditions and analyse which data sources can provide relevant information at different disease stages.

Despite the promising results, our study presents some limitations both from the study and model designs. Firstly, our initial sample exhibited a large amount of missing data, including imaging, clinical, and prognostic labels–including mRS scores. We attempted to address this limitation by imputing the missing data; however, such a method could negatively impact our results by introducing bias towards a specific feature distribution and, consequently, the model results. Further experiments regarding the effects of the imputation are out of the scope of this work but could provide insights into the validity of our approach. Also, the strategy adopted to deal with missing imaging data assumes that the model can learn the impact of the distance from the “real” scan to the predicted time-point. Alternative approaches, such as the synthesis of imaging features, were not tested, which could improve the model results and provide relevant clinical information about intermediate time points where imaging acquisition is impractical.

Our model ignores the effects of TBM dexamethasone treatment in the brain morphology and patients’ outcomes, and their subsequent effect on disease progression and prognosis. The data in this study are from ongoing clinical trials^20,21^, ending in May 2024. Thus, at the time of our study, the authors were blind to the treatment allocation. It is possible that dexamethasone might impact the individual disease progression and models performance. Future work should address treatment effects as a covariate our proposed prognostic tool.

We designed the proposed model as a regression task—continuous prediction of the labels, here the mRS scale—to represent the ordinal nature of the predicted scale. Therefore, the obtained (continuous) predictions were discretised into classes according to a threshold of 0.5. Such an approach could have negatively impacted the model as we did not optimise this threshold to maximise the separation of adjacent classes. Future work should focus on an ordinal model where the predictive classes are discrete while still respecting the ordinal nature of the labels. Alternatively, the threshold could be optimised using the Youden J statistics per class.

Lastly, our population is from Vietnam, with a particular demographic profile, environmental conditions, and comorbidities. Therefore, the validity of our conclusions, alongside the robustness of the model, should be tested in a different population.

## Conclusion

We present a novel machine learning approach aimed at better defining the prognosis of TBM leveraging imaging and clinical data. The proposed model accurately predicted the disease stage for unseen time points for HIV-positive and HIV-negative populations. Despite showing a lower performance in predicting the intermediate stages of the disease, the model was able to predict changes in the disease severity (i.e., anticipating worsening or improvement of the clinical status). Furthermore, from the imaging data, our model identified the typical brain lesions associated with TBM, despite their heterogeneity. The proposed approach demonstrated high potential as a prognostic tool to anticipate severe forms of the disease, enabling timely clinical intervention.

### Supplementary Information

Supplementary Information.

## Data Availability

The datasets used and/or analysed during the current study available from the corresponding author on reasonable request in accordance with the trials data sharing statements. Data are parts of the two randomised control trials: the ACT HIV (identifier NCT03092817) and LAST ACT (identifier NCT03100786).

## Code availability

The code is available upon request to the corresponding author.
