# Supplementary material for: Computer-aided prognosis of tuberculous meningitis combining imaging and non-imaging data
Source: Sci Rep. 2024 Jul 30;14:17581. doi: 10.1038/s41598-024-68308-8 (PMC11289120; doi:10.1038/s41598-024-68308-8)

Computer-aided prognosis of tuberculous meningitis combining imaging and non-imaging data

Liane S. Canas PhD[1], Trinh H.K. Dong[1,3], Daniel Beasley PhD[1], Joseph Donovan PhD[3,4], Jon O. Cleary MBBS-PhD[1,5], Richard Brown PhD[1], Nguyen Thuy Thuong Thuong, PhD[2,3], Phu Hoan Nguyen PhD [2,3], Ha Thi Nguyen[2,3], Prof Reza Razavi[1], Prof Sebastien Ourselin[1], Prof Guy E. Thwaites[2,3], Marc Modat PhD[1] on behalf of the Vietnam ICU Translational Applications Laboratory (VITAL) Investigators

Affiliations

1. School of Biomedical Engineering & Imaging Sciences, King's College London, London, UK.
2. Nuffield Department of Medicine, University of Oxford, Oxford, UK
3. Oxford University Clinical Research Unit, Ho Chi Minh City, Viet Nam
4. London School of Hygiene & Tropical Medicine, London, UK
5. Department of Radiology, Guy's and St. Thomas' NHS Foundation Trust, London, UK

Supplementary Material

Supplementary Methods 3
Committee approvals for the clinical trials: 3
Table S1. Modified Rankin Scale (mRS) interpolation criteria based on clinical assessments. 3
Table S2. Clinical features. List of clinical variables (features) acquired at baseline and during follow-up assessments, and additional features derived from clinical assessment and used as input in the prognosis model. 3
Table S3. Image augmentation.. 4
Table S4. Frequency of output labels on the testing set. 4
Table S5. Model performance when predicting the mRS grade. 4
Figure S1. Flowchart of subjects with valid imaging and clinical data included in the study. 5
Figure S2. Distribution of MRS classes in testing set. 5

Supplementary Methods

Committee approvals for the clinical trials:

A. Ethical approvals for the ACT HIV trial (NCT03092817), with approval numbers, were as follows:

1. The Oxford Tropical Research Ethics Committee: 36-16

2. The ethical committee of the Hospital for Tropical Diseases: 14/HDDD

3. The ethical committee of Pham Ngoc Thach Hospital for Tuberculosis and Lung Disease: 1033/ HDDD-PNT

4. The ethical committee of Dr. Cipto Mangunkusumo National Reference Hospital: LB.02.01/X.2/658/2017

5. The ethical committee of Persahabatan National Respiratory Referral Hospital: 12/KEPK-RSUPP/ 02 /2018

6. The Vietnam Ministry of Health: 108/CN-BDGDD

7. Fakultas Kedokteran Universitas Indonesia (FKUI). Also known as Faculty of Medicine Universitas Indonesia: 52/UN2.F1/ETIK/2017

8. Badan Pengawasan Obat dan Makanan (BPOM). Also known as Indonesian National Agency of Drug and Food Control (NADFC): B-PN.01.06.313.08.17.3791

B. Ethical approvals for the LAST ACT trial (NCT03100786) were as follows:

The trial protocol has been approved by the Oxford Tropical Research Ethics Committee, the Ethics Committees of the Hospital for Tropical Diseases Pham Ngoc Thach Hospital and the Vietnam Ministry of Health.

Table S1. Modified Rankin Scale (mRS) interpolation criteria based on clinical assessments. The mRS is interpolated when missing (scenarios) based on other clinical information available at the time of imaging and clinical data acquisition.

Scenarios	Criteria	MRS interpolated value
I.	An adverse event resulted in the subject's death within 30 days after imaging data acquisition.	6
II.	The imaging data is acquired prior to the first evaluation of MRS, within 30 days of the first MRS assessment.	First mRS collected
III.	Imaging or clinical data was collected during the disease without MRS assessment.	The closest mRS collected.

Table S2. Clinical features. List of clinical variables (features) acquired at baseline and during follow-up assessments, and additional features derived from clinical assessment and used as input in the prognosis model.

Feature	Description	Time of acquisition	Variable type
Sex	Gender at birth: male (0) or female (1).	Baseline assessment	Binary
Body mass index (BMI)	BMI (kg/m^2) is computed using the weight and height of the subject.	Baseline assessment	Continuous
Age	Age, in years, is computed based on the difference between the year of birth and the year of the study (2022).	Baseline assessment	Continuous
HIV status	Yes (1 - positive HIV) or No (0 - Negative HIV).	Baseline assessment	Binary
Known HIV year	Year of diagnosis.	Baseline assessment	Continuous
Neuro day	New neurological events.	Follow-up assessment	Continuous
Cranial Palsy Present	Does the patient have cranial nerves palsy? Yes (1) or No (0)	Baseline assessment	Binary
Temperature	The highest temperature in the last 24 hours.	Baseline and Follow-up assessment	Continuous
CSF cyto white cells	Cytology test: White blood cell count.	Follow-up assessment	Continuous
CSF cyto lymp.	Cytology test: Proportion of lymphocytes.	Follow-up assessment	Continuous
CSF bio protein	Biochemistry test: CSF Protein.	Follow-up assessment	Continuous
CSF bio glucose	Biochemistry test: CSF Glucose.	Follow-up assessment	Continuous
Glasgow Coma Scale (GCS) total	The sum of GCSs, including eye-opening, motor, and verbal scores.	Follow-up assessment	Continuous
Study day mRS	Time (in days) between baseline and current follow-up assessment.	Follow-up assessment	Continuous
Time since scan	Time (in days) between image data acquisition and current follow-up assessment.	Follow-up assessment	Continuous

Table S3. Image augmentation. List of imaging transformations used for data augmentation. Probability defines the probability of an augmentation being applied to each image during model training.

Augmentation	Description	Probability
Scale Intensity	Intensity scale per image to ensure comparative range across all images.	1
Resize	Resize the images to an isotropic lower resolution – easier and faster training.	1
Random Gaussian Noise	Add Gaussian noise to the image.	0.5
Random Bias Field	Random bias field augmentation for MR images.	0.5
Gibbs Noise	The transform applies Gibbs noise to 2D/3D MRI images.	0.5
Rand Affine	Affine transformations are applied in rotate, shear, translate, and scale order.	0.5

Table S4. Frequency of output labels on the testing set. The frequency of output labels is computed across all time points (multiple time points per patient). Frequency for mRS scores and disease progression labels (decrease, stable, increase) in the testing set for all populations (HIV-positive and HIV-negative), and subpopulations. *: Random guessing is defined here as the probability of each label in the all sample ($rg_k = \frac{N_k}{\sum_{k=0}^{K} N_k}$ with rg_k encoding the random guessing for a *k* class, with *K* = 5 for mRS score prediction or *K*=2 for disease progression, and *N* encodes samples per class *k*.)

	All populations		HIV-positive		HIV-negative	
**mRS score**	*Number of samples*	*Random guessing**	*Number of samples*	*Random guessing**	*Number of samples*	*Random guessing**
0	1086	0.45	369	0.39	717	0.49
1	642	0.26	209	0.22	433	0.30
2	186	0.08	108	0.11	78	0.05
3	155	0.06	83	0.09	66	0.04
4	99	0.04	73	0.08	26	0.02
5	256	0.11	109	0.11	147	0.10
**Disease progression**	*Number of samples*	*Random guessing**	*Number of samples*	*Random guessing**	*Number of samples*	*Random guessing**
Decrease	396	0.14	175	0.14	220	0.14
Stable	2242	0.78	992	0.78	1250	0.77
Increase	254	0.09	108	0.08	146	0.09

Table S5. Model performance when predicting the mRS grade. Model evaluation in the testing set using the best-performing model in the validation set. All data refer to the full population, including HIV-p and HIV-n. MCC: Matthew correlation score. FS: Full sequence. LTP: last time-point (outcome). MV-PM: Multivariate prognosis model[9] optimised in OUCRU dataset aiming the prediction of mRS scale.

		**Balanced accuracy**		**MCC**		**Recall weighted**		**Precision weighted**	
	Model	*FS*	*LTP*	*FS*	*LTP*	*FS*	*LTP*	*FS*	*LTP*
All Data (N=39)	MV-PM	0.255	0.276	0.087	0.101	0.413	0.590	0.320	0.453
	Ours - Clinical	0.462	0.451	0.381	**0.460**	0.533	**0.692**	**0.617**	**0.783**
	Ours -Imaging	0.335	0.245	0.223	0.226	0.372	0.436	0.487	0.705
	Ours – C&I	**0.552**	**0.471**	**0.399**	0.337	**0.542**	0.487	0.605	0.741
HIV-p patients (N=19)	MV-PM	0.355	0.300	0.217	0.174	0.361	0.375	0.442	0.458
	Ours -Clinical	0.558	0.350	**0.555**	0.295	**0.646**	**0.500**	**0.714**	**0.500**
	Ours -Imaging	0.299	0.350	0.286	**0.333**	0.437	0.500	0.446	0.563
	Ours -C&I	**0.610**	**0.400**	0.514	0.259	0.589	0.250	0.662	0.150
HIV-n patients (N=20)	MV-PM	0.166	0.190	0.064	0.065	0.432	0.645	0.215	0.452
	Ours -Clinical	0.376	0.531	0.298	**0.513**	0.490	**0.742**	0.514	0.723
	Ours -Imaging	0.344	0.211	0.187	0.196	0.348	0.419	0.502	0.758
	Ours -C&I	**0.477**	**0.558**	**0.350**	0.365	**0.524**	0.548	**0.593**	**0.770**

Figure S1. Flowchart of subjects with valid imaging and clinical data included in the study. Subjects from NCT03092817 and NCT03100786 trials with MRI data - T1w – were assessed for valid imaging data through a visual quality control (QC) of the T1w scans. Subjects with no valid imaging data at any time point were excluded. Subjects were further assessed according to the following inclusion criteria (*): (1) having at least one imaging time-point, (2) having baseline clinical evaluation and imaging data within 30 days of study entry, and (3) having at least one follow-up clinical assessment during the trial (24 months for HIV-n and 12 months for HIV-p participants). Subjects failing with at least one of the inclusion criteria were further excluded. Red arrows and boxes encode excluded subjects, blue encodes HIV-p subsample and green denotes HIV-n subsample.

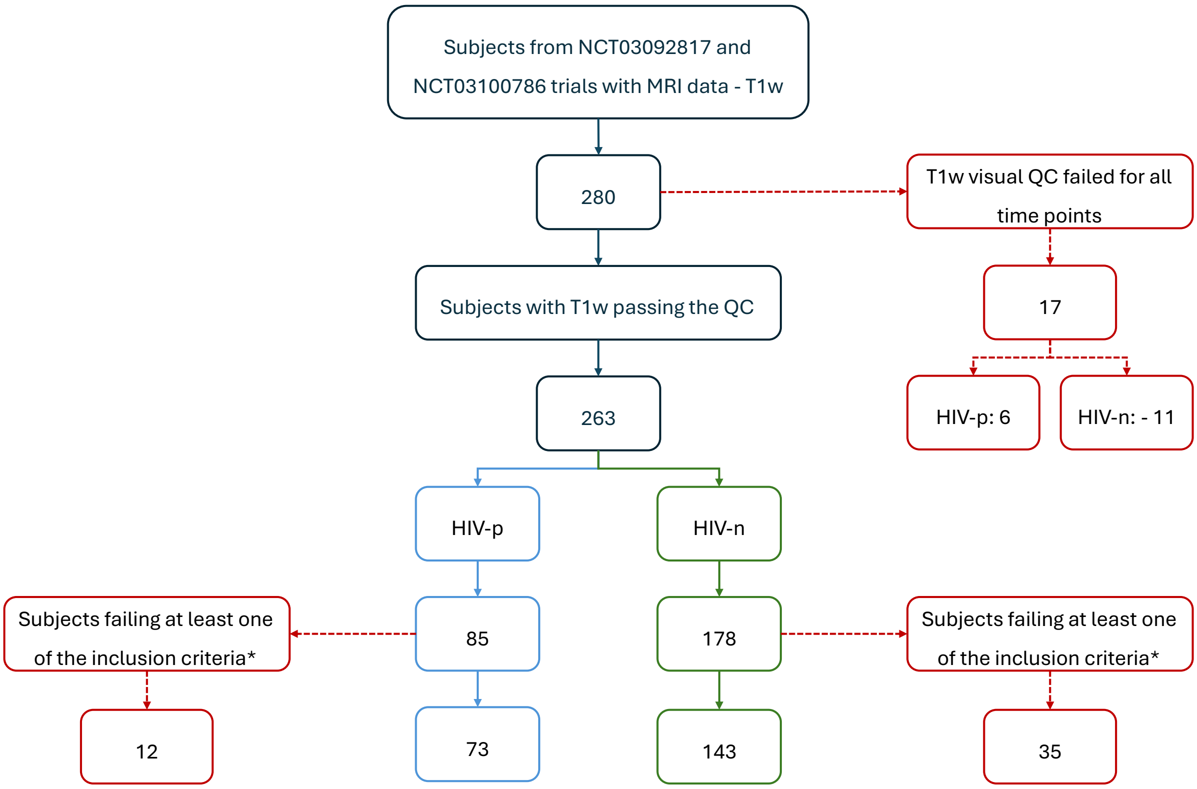

Figure S2. Distribution of MRS classes in testing set.

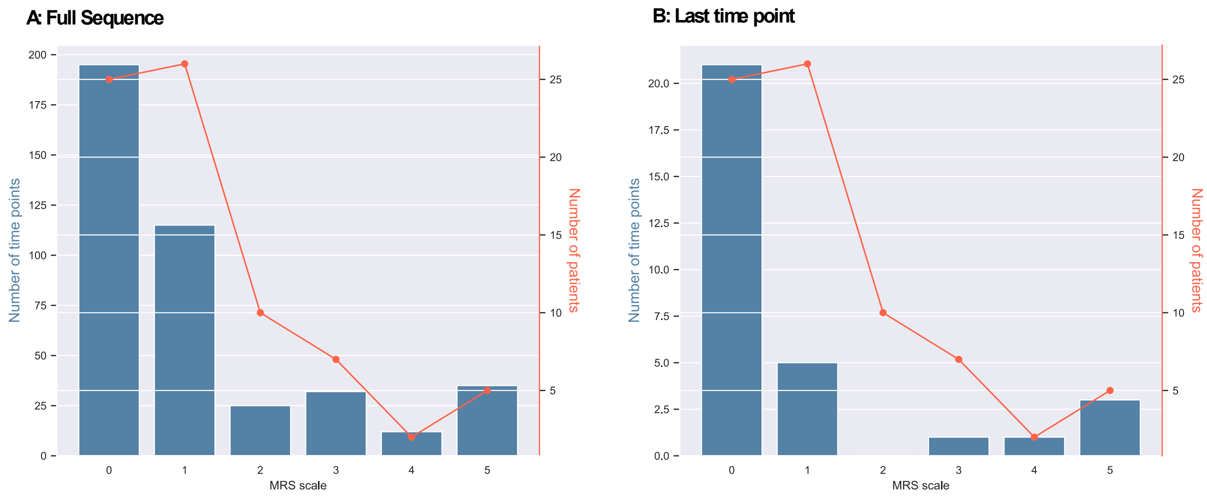

Supplement: Supplementary file 1 — Supplementary Information. [file 41598_2024_68308_MOESM1_ESM.pdf]
